# Supplementary material for: Comparison between Capillary and Serum Lactate Levels in Predicting Short-Term Mortality of Septic Patients at the Emergency Department
Source: Int J Mol Sci. 2023 May 23;24(11):9121. doi: 10.3390/ijms24119121 (PMC10252479; doi:10.3390/ijms24119121)
Supplement: Supplementary file 1 [file ijms-24-09121-s001.zip › ijms-2400505-Supplementary.pdf]

**Supplementary Materials:**

**Supplementary Figure S1.** Bland-Altman method showing that the differences between CLs and SLs assays are within the 95% limits (191 out of 199).

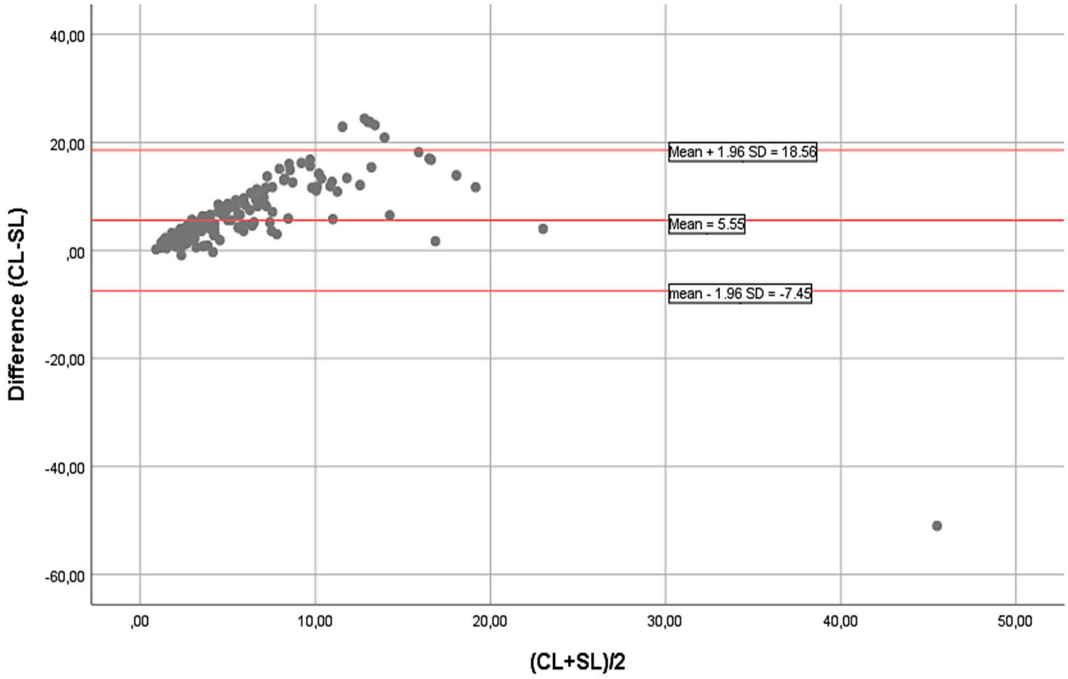


**Supplementary Figure S2**: ROC curves illustrating the discrimination ability of CL, SL, NEWS and SOFA in assessing 48-hour (panel A) and 7-day mortality (panel B).

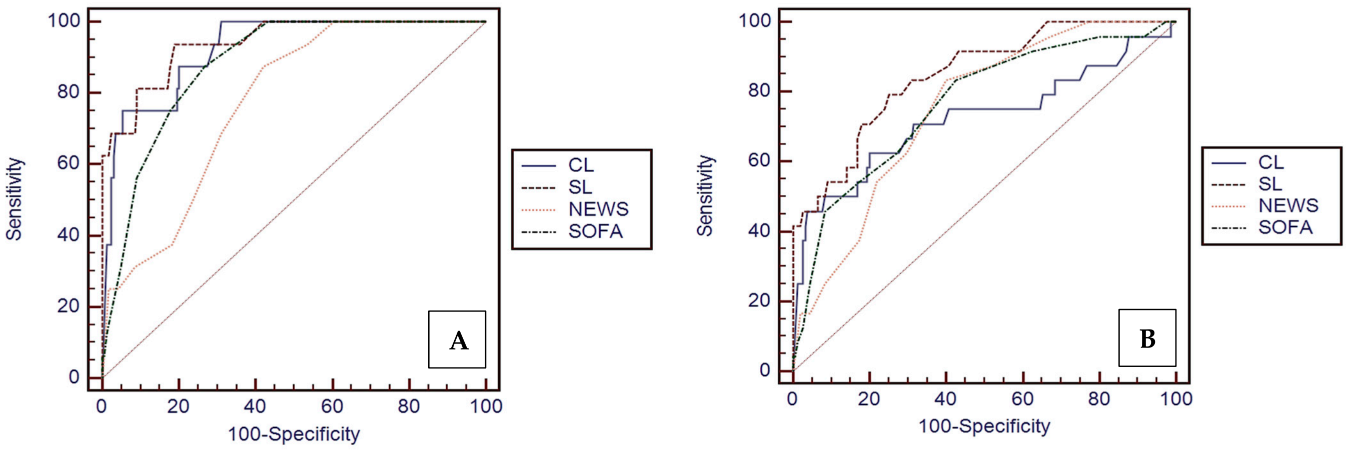


*Footnotes.* Panel A: CL AUC 0.924, 95%CI 0.875-0.958; SL AUC 0.941, 95%CI 0.896-0.970; NEWS AUC 0.773, 95%CI 0.707-0.832; SOFA AUC 0.879, 95%CI 0.823-0.923. Panel B CL AUC 0.724, 95%CI 0.8652-0.788; SL AUC 0.848, 95%CI 0.787-0.898; NEWS AUC 0.751, 95%CI 0.681-0.812; SOFA AUC 0.766, 95%CI 0.697-0.826.

**Supplementary Table S1.** Correlation analysis among main variables.

| | | CLs | NEWS | SOFA | SLs | Age | SBP |
|---|---|---|---|---|---|---|---|
| CLs | Correlation coefficient | 1,000 | ,359 | ,412 | ,497 | -0,101 | -0,105 |
| | *p* | | <0,001 | <0,001 | <0,001 | 0,183 | 0,166 |
| NEWS | Correlation coefficient | ,359 | 1,000 | ,467 | ,422 | 0,112 | -,485 |
| | *p* | <0,001 | | <0,001 | <0,001 | 0,140 | <0,001 |
| SOFA | Correlation coefficient | ,412 | ,467 | 1,000 | ,389 | 0,051 | -,193 |
| | *p* | <0,001 | <0,001 | | <0,001 | 0,498 | 0,010 |
| SLs | Correlation coefficient | ,497 | ,422 | ,389 | 1,000 | -0,091 | -,253 |
| | *p* | <0,001 | <0,001 | <0,001 | | 0,227 | 0,001 |
| Age | Correlation coefficient | -0,101 | 0,112 | 0,051 | -0,091 | 1,000 | 0,061 |
| | *p* | 0,183 | 0,140 | 0,498 | 0,227 | | 0,421 |
| SBP | Correlation coefficient | -0,105 | -,485 | -,193 | -,253 | 0,061 | 1,000 |
| | *p* | 0,166 | <0,001 | 0,010 | 0,001 | 0,421 | |

**Supplementary Table S2.** Sensitivity analysis on patients < 80 and ≥ 80 years assessing the risk of 48-hour mortality.

| | < 80 years | | | ≥ 80 years | | |
|---|---|---|---|---|---|---|
| | OR | 95% CI | *p* | OR | 95% CI | *p* |
| CLs ≥ 16.8 mmol/L | 179.313 | 4.212-7633.634 | 0.007 | 16.459 | 2.395-113.096 | 0.004 |
| NEWS | 1.414 | 0.770-2.599 | 0.264 | 1.148 | 0.791-1.665 | 0.468 |
| SOFA | 1.217 | 0.670-2.212 | 0.519 | 1.895 | 1.108-3.239 | 0.019 |
